# Association of Continuously Measured Vital Signs With Respiratory Insufficiency in Hospitalized COVID-19 Patients: Retrospective Cohort Study

**DOI:** 10.2196/40289

**Published:** 2022-11-23

**Authors:** Harriet M R van Goor, Lisette M Vernooij, Martine J M Breteler, Cor J Kalkman, Karin A H Kaasjager, Kim van Loon

**Affiliations:** 1 Department of Anesthesiology University Medical Center Utrecht Utrecht Netherlands

**Keywords:** continuous monitoring, vital sign monitoring, COVID-19, general ward, vital sign, monitoring, respiration, data, respiratory insufficiency, cohort study, respiratory rate, heart rate, oxygen, clinical

## Abstract

**Background:**

Continuous monitoring of vital signs has the potential to assist in the recognition of deterioration of patients admitted to the general ward. However, methods to efficiently process and use continuously measured vital sign data remain unclear.

**Objective:**

The aim of this study was to explore methods to summarize continuously measured vital sign data and evaluate their association with respiratory insufficiency in COVID-19 patients at the general ward.

**Methods:**

In this retrospective cohort study, we included patients admitted to a designated COVID-19 cohort ward equipped with continuous vital sign monitoring. We collected continuously measured data of respiratory rate, heart rate, and oxygen saturation. For each patient, 7 metrics to summarize vital sign data were calculated: mean, slope, variance, occurrence of a threshold breach, number of episodes, total duration, and area above/under a threshold. These summary measures were calculated over timeframes of either 4 or 8 hours, with a pause between the last data point and the endpoint (the “lead”) of 4, 2, 1, or 0 hours, and with 3 predefined thresholds per vital sign. The association between each of the summary measures and the occurrence of respiratory insufficiency was calculated using logistic regression analysis.

**Results:**

Of the 429 patients that were monitored, 334 were included for analysis. Of these, 66 (19.8%) patients developed respiratory insufficiency. Summarized continuously measured vital sign data in timeframes close to the endpoint showed stronger associations than data measured further in the past (ie, lead 0 vs 1, 2, or 4 hours), and summarized estimates over 4 hours of data had stronger associations than estimates taken over 8 hours of data. The mean was consistently strongly associated with respiratory insufficiency for the three vital signs: in a 4-hour timeframe without a lead, the standardized odds ratio for heart rate, respiratory rate, and oxygen saturation was 2.59 (99% CI 1.74-4.04), 5.05 (99% CI 2.87-10.03), and 3.16 (99% CI 1.78-6.26), respectively. The strength of associations of summary measures varied per vital sign, timeframe, and lead.

**Conclusions:**

The mean of a vital sign showed a relatively strong association with respiratory insufficiency for the majority of vital signs and timeframes. The type of vital sign, length of the timeframe, and length of the lead influenced the strength of associations. Highly associated summary measures and their combinations could be used in a clinical prediction score or algorithm for an automatic alarm system.

## Introduction

Hypoxic respiratory failure is a common complication of COVID-19, caused by severe viral pneumonia or concomitant pulmonary embolism [1,2]. Respiratory deterioration can occur suddenly and sometimes without signs of dyspnea [3,4], which complicates detection. Tools to assess the vital instability of patients more frequently could help to detect respiratory deterioration in a timely manner. Currently, most hospitals use a form of early warning score as a “track-and-trigger” system at the general ward to aid health care professionals in the detection of deterioration [5]. Early warning scores can vary from scores with a few physiological parameters, such as the Modified Early Warning Score [6], to machine-learning algorithms including baseline patient characteristics and laboratory results [7]. However, these models use intermittent measurements to update their prediction, and are therefore limited by the frequency of spot-check measurements and laboratory tests.

An alternative strategy to improve the early detection of deterioration could be continuous monitoring including assessment of vital signs. Continuous monitoring can be beneficial in two ways. First, trends in vital signs over time have been shown to have higher predictive accuracy than isolated vital sign values when incorporated in prediction models [8,9]. With continuous monitoring, trends in vital signs are available at any point in time, and can therefore be used to make up-to-date predictions more frequently. Unfortunately, prediction models using continuously measured vital sign data at the general ward are not yet readily available for clinical use. A second benefit of continuous monitoring is that it enables health care professionals to access the real-time vital sign status of a patient remotely, and to use this information in clinical decision-making [10]. However, nurses and physicians at low-care wards are usually not used to, or trained in, evaluating continuous vital sign data [11]. Current practice is therefore mostly based on experience and expert opinion. Knowledge of “what to look for” in vital sign trends could aid nurses and physicians to interpret continuously measured data in a meaningful way.

In this study, we assessed several measures to summarize continuously measured vital sign data, and evaluated their association with respiratory insufficiency in COVID-19 patients admitted to the general ward. We aimed to find summary measures that could be clinically helpful to recognize respiratory deterioration early, which might further be useful to incorporate into an algorithm for automatic alarming.

## Methods

### Population and Setting

At the beginning of April 2020, a continuous wireless system for vital sign monitoring was introduced at the COVID-19 cohort ward of the tertiary hospital University Medical Center Utrecht, Utrecht, the Netherlands. This system recorded heart rate (HR) and respiratory rate (RR) using a validated wireless patch sensor [12] (Biosensor Voyage, Philips Electronics Netherlands BV), and peripheral oxygen saturation (SpO2) via a finger pulse oximeter (EarlyVue VS30, Philips Electronics Netherlands BV) every 30 seconds approximated over the past 30 seconds. Data were stored in the software program AnStat (CarePoint Nederland BV, Ede, the Netherlands). Pulse oximeters were delivered later than the wearable sensors (end of May 2020). We included patients from April 2020 until March 1, 2021. Patients were included if they were ≥18 years old, diagnosed with COVID-19, and continuously monitored during their admission at the study ward (either with the biosensor, pulse oximeter, or both). Patients with a pacemaker did not receive a sensor since RR measurements are unreliable in paced rhythms. All continuously measured data were available in real time for hospital staff, without a predefined protocol on how to use continuously measured data or how to detect respiratory insufficiency. The protocol in use for detecting deterioration in general was the National Early Warning Score (NEWS) 2 [13]. The updated Charlson Comorbidity Index was used to assess the baseline risk of 1-year mortality [14].

### Ethical Considerations

The study was conducted according to the principles of the Declaration of Helsinki and the General Data Protection Regulation [15,16]. Ethical review was waived by the medical ethical committee Utrecht (MEC-20-365). Patients were offered the chance to opt out of retrospective data analyses during hospital registration and again at hospital discharge, according to the institutional protocol. The data were previously used in a study of circadian rhythm in continuously measured vital signs [17].

### Primary Endpoint

The primary endpoint was respiratory insufficiency, which we defined as the need for 15 L/min oxygen, high-flow nasal oxygen therapy, or mechanical ventilation, whichever came first. We did not deem intensive care unit (ICU) admission or death to be a suitable endpoint since a substantial portion of the population had treatment restrictions preventing them from receiving cardiac resuscitation, mechanical ventilation, and/or ICU admission. Moreover, high-flow nasal oxygen therapy was also given at the general ward, since ICU beds were not always available. The first documentation of the endpoint in the electronic patient record was used as the time point for respiratory insufficiency.

### Data Selection

**Figure 1:**
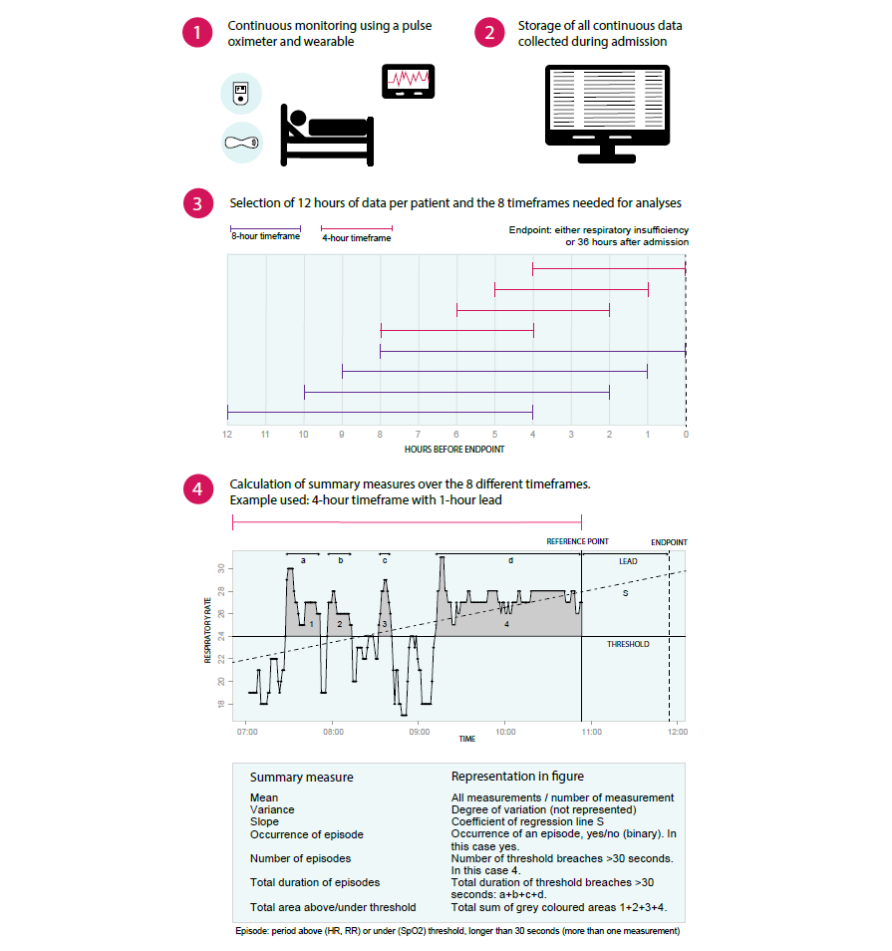
Data selection for continuous heart rate (HR), respiratory rate (RR), and oxygen saturation (SpO2).

For each patient, we selected 12 hours of continuous vital sign data. For patients who became respiratory-insufficient, we selected the 12 hours of data prior to the onset of respiratory insufficiency. The distribution of the timing of reaching the endpoint in our cohort was approximately 40 hours after starting monitoring, with a right-skewed distribution (ie, several cases reached respiratory insufficiency before the 40-hour point). For patients who did not reach this endpoint, we selected the data from 24 hours up to 36 hours after admission (Figure 1). We chose this window since the majority of patients were connected to the monitoring system within 24 hours after admission. Moreover, the median time until respiratory insufficiency in our cohort was 40.6 hours (IQR 22.6-70.4) after starting monitoring with a right-skewed distribution. By selecting 24-36 hours after starting monitoring for the control group, the timing for the timeframes for both the endpoint and control group were fairly similar. Potential artefacts (RR<1/min or >80/min, HR <30/min or >280/min, SpO2<50%, and large abrupt changes in RR [>20 breaths/min] and HR [>25 beats/min] that lasted for less than 2 minutes) were removed. For each patient, we divided the selected data of 12 hours into 8 different timeframes of either 4 or 8 hours long (Figure 1). We chose these lengths because they clinically correlate with the length of a usual half and full shift of hospital professionals. In addition, we shifted timeframes either 0, 1, 2, or 4 hours from the end of the selected 12-hour data window (the “lead”) (Figure 1). For example, with a lead of 4 hours, we assessed whether associations could already be observed 4 hours before the onset of respiratory insufficiency. To handle missing data, timeframes were only included if the first measurement of a timeframe was within 30 minutes of the start of the timeframe and the last measurement was within the last 30 minutes of the timeframe. We did this to avoid selection of timeframes that were actually smaller than assumed due to missing data (eg, if a timeframe of 8 hours only contains 5 hours of data, it is not actually an 8-hour timeframe but rather a 5-hour timeframe).

### Selection of Summary Measures

As the continuous monitoring of vital signs provides measurements twice every minute, we summarized the continuously measured data into “summary measures.” Summary measures are either unrelated to a certain threshold (eg, the mean HR) or related to a threshold (eg, duration of HR>89/min). For all threshold-related variables, we chose three thresholds per vital sign. Thresholds for HR were based on the three upper thresholds for tachycardia of the NEWS2: >90/min, >110/min, and >130/min [13]. For tachypnea, we used the upper two levels of the NEWS2, >20/min and >24/min, and added a third level, >30/min, since the first upper threshold of the NEWS2 would be met by almost every COVID-19 patient. We used the lower two levels of SpO2, <94% and <92%, and added <90% for similar reasons. We chose not to include bradycardia or bradypnea since these were very uncommon as signs of respiratory insufficiency in our population. An episode was defined as more than one measurement (longer than 30 seconds) above or under a certain threshold. Initially, we defined 12 summary measures based on the literature and clinical reasoning [8,18] (Multimedia Appendix 1). Correlation plots of these summary measures showed high correlations between several measures. The summary measure “standard deviation” showed high correlation with “variance” and was therefore eliminated. “Mean duration,” “maximum duration,” and “total duration” above/under the threshold were highly correlated; therefore, we only included “total duration.” A similar choice was made for area above/under the threshold. Ultimately, we selected seven summary measures for analysis: three summary measures unrelated to a threshold and four summary measures related to a threshold (Figure 1).

### Statistical Analysis

Baseline characteristics are described for both cohorts. For every patient, all selected summary measures were calculated for the eight timeframes. To investigate the crude association between each of the summary measures and the development of respiratory insufficiency, univariable logistic regression was performed. Effect estimates are reported as odds ratios (ORs) with accompanying CIs. Since the mean and slope of SpO2 have a negative relationship with the endpoint (a decrease in oxygen saturation is associated with the endpoint instead of an increase), the inverse effect estimate is reported for these two summary measures of SpO2. As the selected summary measures had different units of measurement (eg, duration in minutes, area above the threshold in /min×duration or %×duration), we could not directly compare their associations with each other based on crude ORs. Therefore, we used standardized odds ratios (sORs) to compare the association of different summary measures with respiratory insufficiency on a similar magnitude. Standardized summary measures for each patient were calculated using the formula Z=(x–μ)/σ, where Z is the newly computed standardized value, x is the summary measure for a particular patient, μ is the mean of the same summary measure for all patients in this timeframe, and σ is the standard deviation of all patients in the respective timeframe. With these standardized measures, the sORs were calculated. For example, an sOR of 2 for a certain summary measure means that if the standard deviation of this measure increases by one, the association with respiratory insufficiency increases by two.

To take multiple testing into account, we tested against a *P* value of .01 for all aforementioned analyses. Bonferroni adjustment was deemed too conservative since the chosen summary measures are highly dependent on each other. We used R software version 4.0.3 (R foundation for Statistical Computing, Vienna, Austria 2021) for all analyses.

## Results

### Cohort Characteristics

**Figure 2:**
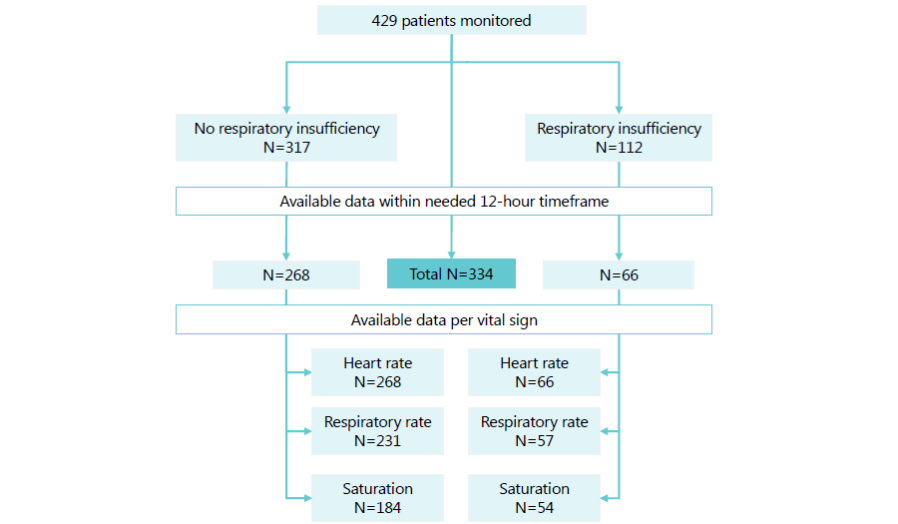
Flowchart of patient inclusion based on data availability.

The description of the cohort is provided in Table 1. Of the 429 patients that were monitored, 334 were included for analysis (Figure 2), 66 (19.8%) of whom developed respiratory insufficiency. These patients more often had pulmonary embolism, ICU and medium-care unit admissions, treatment restrictions, and had higher mortality rates (Table 1). Two patients who did not experience respiratory insufficiency were shortly admitted to a high-care unit during monitoring: one patient required monitoring for severe hypokalemia, and the other patient suffered a stroke and was admitted for thrombolysis. All patients had available HR data. The sample of patients with RR data was smaller (n=288) since the sensor had to be calibrated to measure RR, which was not always executed immediately. Due to late delivery and noncompliance of patients with the pulse oximeter, SpO2 data were available for only 238 patients. At baseline, these samples differed slightly with respect to the number of patients that received dexamethasone and the number of patients that reached the endpoint (Multimedia Appendix 2). Overall, patients who developed respiratory insufficiency had a higher occurrence of threshold breaches, and spent more time above thresholds for HR and RR and under the thresholds for SpO2 in both the 4-hour and 8-hour timeframes (Multimedia Appendix 3). The mean number of measurements per hour was 79 for 4-hour timeframes and 74 for 8-hour timeframes. Of all timeframes used, 98.8% contained at least 120 measurements.

**Table 1:** Baseline characteristics and summary of available data.

Characteristic		All (N=334)		No respiratory insufficiency (n=268)	Respiratory insufficiency (n=66)
Age (years), median (IQR)		65 (55.3-73.8)		63 (55-72)	67 (60-75)
Male sex, n (%)		207 (62.0)		168 (62.7)	39 (59.1)
Charlson Comorbidity Index, median (IQR)		0 (0-1)		0 (0-1)	0 (0-2)
Dexamethasone during admission, n (%)		262 (78.4)		208 (77.6)	54 (81.8)
Diagnosed with pulmonary embolism, n (%)		24 (7.2)		14 (5.2)	10 (15.2)
Treatment restrictions^a^, n (%)		91 (27.2)		62 (23.1)	29 (43.9)
Length of hospital stay, median (IQR)		7 (5-12)		6.5 (4.8-10)	13 (9-24)
ICU^b^ or MCU^c^ admission, n (%)^d^		57 (17.1)		30 (11.2)	27 (40.9)
Mortality, n (%)		23 (6.9)		2 (0.7)	21 (31.8)
**Heart rate**					
	Patients with available data, n (%)	334 (100.0)	268 (100.0)		66 (100.0)
	Duration per patient (hours), mean (SD)	11.7 (1.1)	11.8 (0.8)		11.4 (1.7)
**Respiratory rate**					
	Patients with available data, n (%)	288 (86.2)	231 (86.2)		57 (86.4)
	Duration per patient (hours), mean (SD)	11.6 (1.4)	11.7 (1.1)		11.1 (2.1)
**Peripheral oxygen saturation**					
	Patients with available data, n (%)	238 (71.3)	184 (68.7)		54 (81.8)
	Duration per patient (hours), mean (SD)	11.4 (1.4)	11.5 (1.1)		10.9 (2.1)

^a^Treatment restrictions: no resuscitation, no ventilation, and/or no ICU admission.

^b^ICU: intensive care unit.

^c^MCU: medium-care unit.

^d^If a patient was monitored after ICU admission and did not reach the endpoint while being monitored, they were included in the “no respiratory insufficiency” group.

### Association of Summary Measures with Respiratory Insufficiency

Since the highest crude ORs were observed in the 4-hour timeframe without a lead, we have outlined the results of the analysis for this timeframe in Table 2 and Table 3 for measures without and with a threshold, respectively. The summary measure with the highest crude OR was the occurrence of RR>24/min (Table 3). For many summary measures of RR and SpO2, CIs were extremely wide, in particular in summary measures that were threshold-dependent and for which the threshold was breached by the majority of the patients in an outcome group. For example, a threshold breach for SpO2 of <90% occurred in all but one case of patients who experienced respiratory insufficiency, leading to a 99% CI of 1.64-915.0. This phenomenon was also seen in other timeframes. When comparing the standardized ORs in the 4-hour timeframe without a lead, we found stronger associations for RR and SpO2 than for HR. The mean showed a strong association for all three vital signs, with an sOR of 2.59 (99% CI 1.74-4.04) for HR, 5.05 (99% CI 2.87-10.03) for RR, and 3.16 (99% CI 1.78-6.26) for SpO2.

**Table 2:** Results of univariable, nonstandardized, analyses for 4-hour timeframes without a lead for measures with no threshold.

Parameter	Mean^a^		Slope^b^		Variance^c^	
	OR^d^ (99% CI)	*P* value	OR (99% CI)	*P* value	OR (99% CI)	*P* value
Heart rate	1.06 (1.03-1.08)	<.001	1.02 (0.99-1.00)	.64	1.00 (0.99-1.00)	.99
Respiratory rate	1.44 (1.27-1.67	<.001	1.30 (0.88-1.93)	.09	1.09 (1.01-1.17)	.003
Oxygen saturation	1.61 (1.27-2.04)	<.001	1.79 (0.90-3.70)	.03	1.21 (1.09-1.38)	<.001

^a^Calculated as /min for heart rate and respiratory rate and as % for oxygen saturation.

^b^Calculated as /min/hour for heart rate and respiratory rate and as %/hour for oxygen saturation.

^c^Calculated as /min^2^ for heart rate and respiratory rate and as %^2^ for oxygen saturation.

^d^OR: odds ratio.

**Table 3:** Results of univariable, nonstandardized, analyses for 4-hour timeframes without a lead for measures with a threshold.

Threshold			Occurrence					Number of episodes					Total duration (min)					Total area above threshold (/10 min)		
			OR^a^ (99% CI)		*P* value		OR (99% CI)			*P* value		OR (99% CI)			*P* value		OR (99% CI)			*P* value
**Heart rate (/min)**																				
	>90	4.50 (1.95-11.8)		<.001		1.12 (1.03-1.23)			<.001		1.01 (1.01-1.01)			<.001		1.00 (1.00-1.01)			<.001	
	>110	2.79 (1.24-6.18)		<.001		1.16 (1.02-1.35)			.005		1.01 (1.00-1.03)			.002		1.00 (1.00-1.01)			.04	
	>130	6.09 (1.56-25.5)		<.001		1.98 (1.04-5.01)			.03		1.01 (1.00-1.04)			.08		1.00 (0.99-1.02)			.37	
**Respiratory rate (/min)**																				
	>20	7.35 (1.03-531.0)		.05		0.89 (0.77-1.01)			.03		1.02 (1.01-1.03)			<.001		1.04 (1.02-1.05)			<.001	
	>24	13.8 (3.68-103.4)		<.001		1.21 (1.09-1.35)			<.001		1.02 (1.01-1.03)			<.001		1.05 (1.03-1.08)			<.001	
	>29	8.53 (3.63-21.2)		<.001		1.59 (1.32-1.98)			<.001		1.02 (1.01-1.04)			<.001		1.07 (1.03-1.13)			<.001	
**Oxygen saturation (%)**																				
	<94	12.6 (4.11-47.7)		<.001		1.30 (1.11-1.56)			<.001		1.05 (1.02-1.09)			<.001		0.80 (0.67-0.91)			<.001	
	<92	12.3 (3.07-94.5)		<.001		1.30 (1.15-1.49)			<.001		1.03 (1.02-1.05)			<.001		0.88 (0.82-0.94)			<.001	
	<90	11.9 (1.64-915.0)		.02		1.09 (1.01-1.19)			.006		1.02 (1.01-1.03)			<.001		0.93 (0.90-0.96)			<.001	

^a^OR: odds ratio.

### Summary Measures for HR

**Figure 3:**
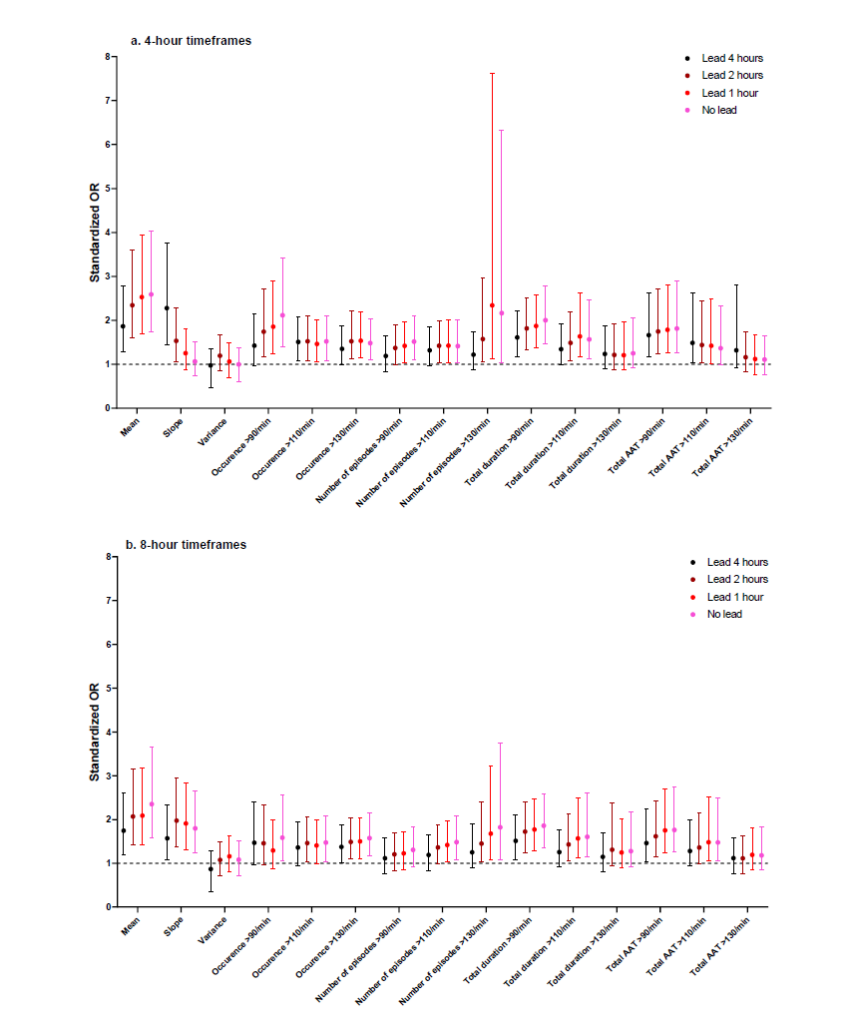
Standardized odds ratios (ORs) for heart rate. AAT: area above threshold.

The highest sORs were observed for mean HR in the 4-hour timeframe without a lead (2.59, 99% CI 1.75-4.04) (Figure 3). Only three summary measures were significantly associated with respiratory insufficiency in all timeframes: the mean, total duration >90/min, and total area above the threshold>110/min. In general, associations were stronger for summary measures in 4-hour timeframes compared with 8-hour timeframes. A notable exception was the slope, for which the associations were low or insignificant in three 4-hour timeframes, but relatively strong in the 8-hour timeframes. Differences in sORs between leads were small for most summary measures.

### Summary Measures for RR

**Figure 4:**
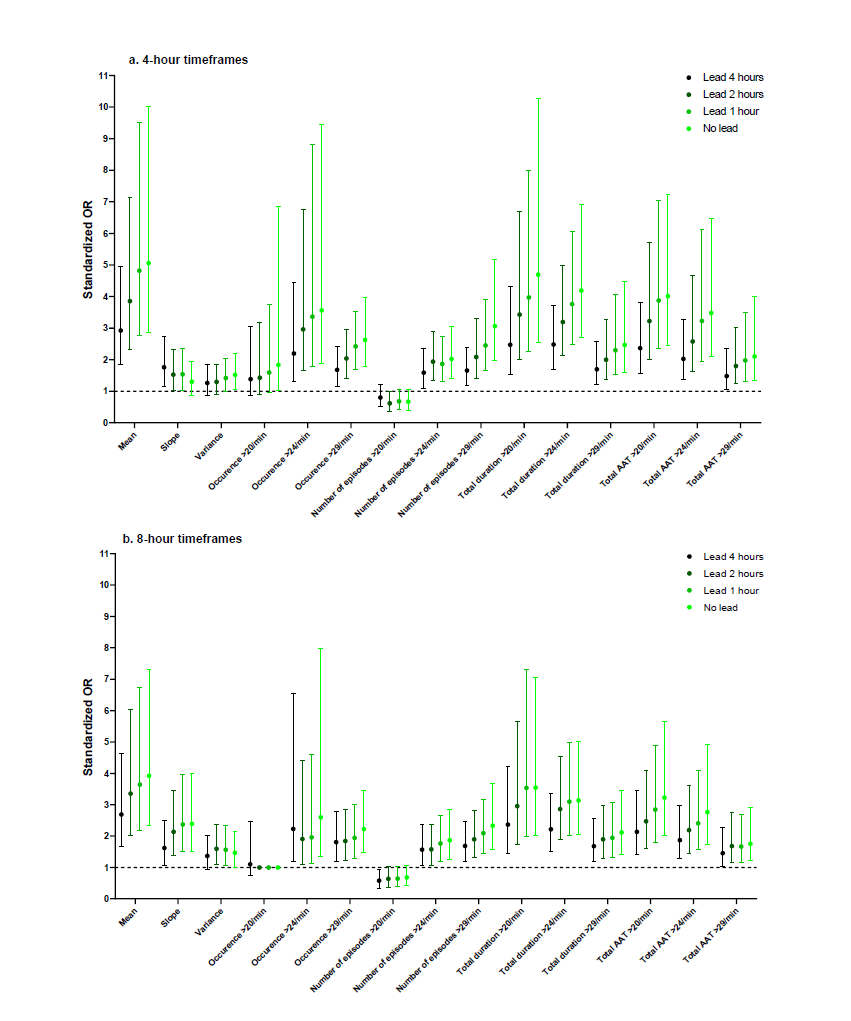
Standardized odds ratios (ORs) for respiratory rate. AAT: area above threshold.

The highest sORs were observed for mean RR (sOR 5.05, 99% CI 2.87-10.03) and the total duration of RR>20/min (sOR 4.69, 99% CI 2.55-10.29) in the 4-hour timeframe without a lead (Figure 4). For most threshold-dependent summary measures, the strength tended to decline when the lead increased, but still reached significance. Summary measures calculated over 4-hour timeframes had stronger associations than those calculated over 8-hour timeframes. Remarkably, the number of episodes >20/min was negatively associated with respiratory insufficiency, likely because to have multiple episodes above 20/min, a patient would also need periods of time with an RR under 20/min, which was not exhibited by the patients showing the most deterioration.

### Summary Measures for Oxygen Saturation

**Figure 5:**
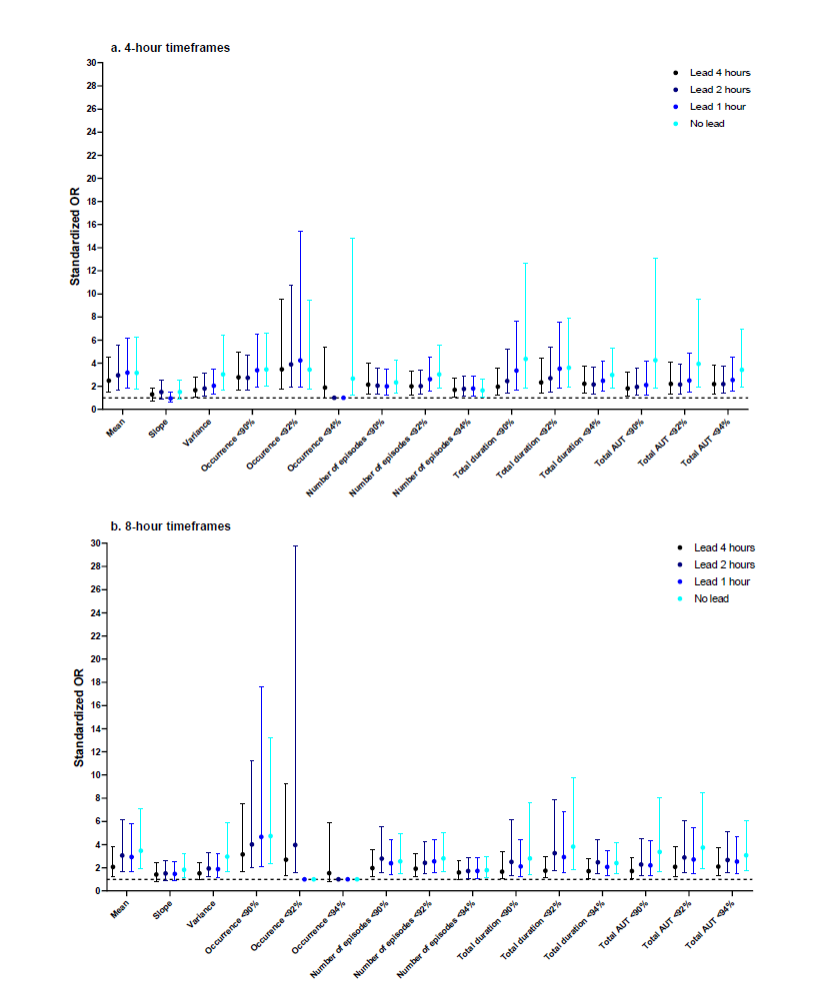
Standardized odds ratios (ORs) for oxygen saturation. AUT: area under threshold.

For SpO2, CIs were generally wider due to the smaller sample size. The strongest association was found in the 8-hour timeframe without a lead, for occurrence of SpO2<90% (sOR 4.74, 99% CI 2.36-13.23) (Figure 5). SpO2 was the only vital sign for which associations were generally slightly stronger in 8-hour timeframes. For many summary measures, the association with respiratory insufficiency was evidently stronger in the timeframes without a lead, especially for variance, total duration, and total area under the threshold. The mean showed a weaker association than some threshold-related summary measures such as occurrence of SpO2<94% in 4-hour timeframes. Nonetheless, the mean was significantly associated with respiratory insufficiency in all timeframes.

## Discussion

### Principal Findings

In this study, we aimed to explore which summary measures for continuously measured HR, RR, and SpO2 data could be helpful in recognizing imminent respiratory insufficiency in COVID-19 patients at the general ward. We found that summary measures over timeframes of continuously measured data close to the endpoint of respiratory insufficiency showed stronger associations than timeframes further removed, and that 4-hour timeframes performed better than 8-hour timeframes. The summary measure “mean” was consistently strongly associated with respiratory insufficiency for all vital parameters. The strength of associations of summary measures depended on the vital sign, timeframe, and lead.

### Comparison With Prior Work

RR has repeatedly been marked as the best discriminator to identify patients at risk for deterioration [19,20]. In our study, we confirmed that RR showed stronger associations with respiratory insufficiency than HR. SpO2 was also strongly associated with respiratory insufficiency, which can partly be explained by the population (COVID-19 patients) and the endpoint (respiratory failure). In a previous study on trends in vital signs of hospitalized patients, Churpek et al [8] used several summary measures for trend analysis of intermittent data to predict clinical deterioration. They found that the slope, mean, and standard deviation were better predictors than the current value for HR and RR, and the mean performed well for SpO2. In our study, we confirmed a strong association of the mean of all three vital parameters with respiratory insufficiency. However, the slope and standard deviation were less informative. This might be caused by differences between intermittently and continuously measured vital signs. Due to the high density of measurements, continuously monitored vital sign data show more variance than intermittent data in our experience, and are more subject to peaks and troughs depending on a patient’s activity level. Akel et al [9] also found that the (intermittently measured) maximum RR and HR were important predictors. We did not use the maximum value, since we expected the maximum value to rely highly on both activity level and outliers (eg, due to coughing or talking), and would therefore not be clinically useful. A recent study did use summary measures for continuously measured vital signs (mean, standard deviation, range, and mean absolute deviation) over 3-hour timeframes [21]. The authors created a machine-learned model of these summary measures along with other data features, and managed to predict complications in postoperative patients with a lead of 12 hours. Unfortunately, this method does not allow for comparison of the value of these different summary measures. In our study, we only used leads up to 4 hours to limit the number of computations. We found that shorter leads led to stronger associations. This might be an obvious finding, as vital instability is often a gradual process of decline, most pronounced at the end when a patient becomes respiratory-insufficient [22]. However, this finding nuances earlier failure-to-rescue statements and illustrates that the information content is less dense 12 hours prior to the event [23,24]. In current clinical practice (and at our study ward) the NEWS2 is often used to detect deterioration [13]. Our thresholds were based on this score. In the NEWS2, more severe threshold breaches receive more points, and thus correspond with a higher risk of poor outcome. From this context, we would have expected summary measures of more severe thresholds to have a stronger association with respiratory insufficiency. Interestingly, this was not the case.

### Methodological Decisions and Limitations

In this exploratory study, we made several methodological decisions that affected the results. First, we chose a cross-sectional method to determine the association of summary models with respiratory insufficiency, by comparing patients who reached the endpoint with those who did not. A longitudinal assessment of risk for respiratory insufficiency (eg, using a dynamic prediction model) might be an approach that is more in line with clinical practice. In a dynamic prediction model, previously recorded data of a patient can be included to update the estimated patient’s risk of developing the outcome of interest at consecutive time points. However, the sample sizes of existing continuous monitoring studies are relatively small and the populations are heterogeneous, which may complicate the development of robust prediction models [10]. Larger studies and open sharing of continuous data might speed up the process of developing and validating such longitudinal dynamic models. A second methodological key decision was to only select summary measures, timeframes, and models that could easily be understood by health care professionals. Hereby, we limit the “black box” effect of complex models, for which the exact computational procedure is opaque [25]. These models might have better predictive accuracy, but are unintelligible for clinical professionals, which makes clinicians reluctant to use and rely on them [25]. For this explorative study, we aimed to increase the understanding of the association between continuous vital signs and deterioration, and therefore we chose a transparent methodology. However, machine-learning models have proven to be more accurate than current practice in several fields of medicine [26]. In predicting deterioration, some studies have shown that machine-learning models outperform “simple” regression models [9,27,28]. In a recent study, a machine-learning model was developed that uses several summary measures of vital signs to predict the deterioration of high-risk patients [21]. Explorative studies such as the present study could provide insight into which summary measures to include into such a machine-learning model [21]. A final addition to a model with continuous monitoring data could be nonvital sign parameters such as the amount of administered oxygen. The combination of administered oxygen with RR and SpO2 has previously shown to accurately predict respiratory insufficiency in COVID-19 patients [29]. Regardless of the type of prediction model or algorithm that is constructed using continuously measured vital sign data, any model should be well calibrated and be externally validated before implementation in clinical practice [30].

### Strengths and Limitations

Beside the above-mentioned methodological considerations, this study has several limitations. We relied on a small convenience sample size, which resulted in limited accuracy, and we were unable to validate our findings in a larger data set. A significant portion of the initial sample had to be excluded owing to lack of continuous monitoring data within the needed 12-hour timeframe for multiple reasons such as loss of connection with the patch, nurses that were not able to or forgot to connect a patient to the system, or patients who reached the endpoint before or within a short time after getting the patch. The exact reasons for these periods of missing data are hard to reconstruct retrospectively. Additionally, there were differences in the number of patients with available data for each vital sign. Patients who were relatively less ill wore the pulse oximeter less often, because they found it annoying, they were mobilizing beyond the reach of the monitor, or the nurse agreed it was no longer necessary to monitor SpO2. The smaller sample size with a relatively high percentage of patients reaching the endpoint might have strengthened the association between SpO2 and respiratory insufficiency. For control patients, we included the 24-36 hours of data following admission to the ward. This was a pragmatic decision, but the choice of timeframes early in the admission period could have influenced the results. Nevertheless, respiratory insufficiency also mostly occurred early in the admission, and therefore the timing of the selected data of the control group and the respiratory insufficiency group was fairly similar. Conclusions can only be drawn for COVID-19 patients. For other patient populations, alternative vital signs and summary measures might be more informative [31]. Since the continuous monitoring system was not implemented alongside standard intermittent monitoring, we could not compare the performance of summary measures with care as usual. All continuous data were visible to the nurses and physicians during the study. The vital sign aberrations they spotted during monitoring will have influenced their decision to start treatment, thus influencing the endpoint. However, staff was limited in the options for additional treatment, and thereby also limited in their influence to avoid the endpoint. Moreover, we do believe the decision to start 15 L/min oxygen therapy, high-flow oxygen therapy, or mechanical ventilation was not solely based on the continuous data but was rather mostly based on the overall clinical condition of the patient. Due to the retrospective nature of the study, we relied on documentation in the electronic patient record to determine the time point of respiratory insufficiency. A prospective design might result in a more accurate estimation of the timing of onset.

### Considerations for Future Research

Summary measures of vital signs that show a strong association with the occurrence of respiratory insufficiency could be helpful in several ways. First, they might be used in a clinical score for direct use by nurses and physicians. For intermittently measured vital signs, the early warning score created both a framework to measure vital stability and a language for nurses to communicate instability to a physician [32]. Nurses are empowered by these aspects of the early warning score. Furthermore, they can use the early warning score to easily package and summarize information about a patient, which helps physicians to prioritize care [32]. For continuous data, no such language or score currently exists to communicate observations of continuously measured vital signs. Summary measures could be used to create such as score. For example, the most strongly associated summary measures could be used to create an easy-to-use prognostic score, or could directly aid nurses in physicians to interpret, summarize, and articulate continuous monitoring data of COVID-19 patients.

Second, summary measures may be useful to be incorporated in an automatic alarm system, especially under circumstances where the nurse-to-patient ratio is low. For example, during a night shift, an alarm system with high predictive accuracy that could detect deteriorating patients that otherwise might have been missed would be valuable [33]. However, clinical scores might not be suitable for automatic alarming. Early warning scores have previously been applied as an alarm system by using the means of continuously monitored vital signs over a short period (eg, 5 minutes) as input values [34,35]. The downside of this strategy is that some thresholds of commonly used early warning scores, such as RR>20/min or HR>90/min in the NEWS2, are easily breached, especially in active patients. In our study, the mean RR for patients who did not experience respiratory insufficiency was 20.5/min; thus, half of all measurements would score a point on the NEWS2. In a recent study, both patients who did and did not experience deterioration met the criteria for a high early warning score if continuous monitoring of vital signs was used [35]. This high number of threshold breaches could, if followed up with an alarm, lead to alarm fatigue [36]. To develop an adequate alarm system, a new predictive model for continuous monitoring data might be more helpful, in which summary measures could be used as input values instead of single threshold breaches. Since such an alarm system can operate in the background and does not have to be used by hospital professionals directly, it would allow for more complexity than the clinical score that is used on the ward. The previously mentioned study by Kristinsson et al [21] is a promising example.

### Conclusions

We explored several possible ways to summarize continuous vital sign data of COVID-19 patients on the general ward. The mean showed a relatively strong association with respiratory insufficiency for HR, RR, and SpO2. Overall, shorter timeframes with smaller leads showed stronger associations. Highly associated summary measures and their combinations could be used in a clinical prediction score or algorithm for an automatic alarm system. 

## Appendix

Multimedia Appendix 1 Considered and selected summary measures.

Multimedia Appendix 2 Baseline characteristics of patients stratified by availability of vital sign data.

Multimedia Appendix 3 Mean summary measures for 4-hour and 8-hour timeframes.

## Abbreviations

HR: heart rate
ICU: intensive care unit
NEWS: National Early Warning Score
OR: odds ratio
RR: respiratory rate
sOR: standardized odds ratio
SpO2: peripheral oxygen saturation

## Data Availability

The data sets analyzed during this study are stored in the data repository DataverseNL [37]. Metadata are publicly available. The data themselves are not publicly available due to their privacy-sensitive nature, but can be requested via DataverseNL.
