# Supplementary material for: Association of Continuously Measured Vital Signs With Respiratory Insufficiency in Hospitalized COVID-19 Patients: Retrospective Cohort Study
Source: Interact J Med Res. 2022 Nov 23;11(2):e40289. doi: 10.2196/40289 (PMC9688258; doi:10.2196/40289)
Supplement: Multimedia Appendix 1 [file ijmr_v11i2e40289_app1.docx]

| Multimedia Appendix 1. Considered and selected summary measures. | | |
| --- | --- | --- |
| Summary measure | Threshold related | Selected for analysis |
| Mean | No | Yes |
| Standard deviation | No | No |
| Variance | No | Yes |
| Slope | No | Yes |
| Occurrence of episode | Yes | Yes |
| Number of episodes | Yes | Yes |
| Total duration of episodes | Yes | Yes |
| Maximum duration of episode | Yes | No |
| Mean duration of episode | Yes | No |
| Total area above/under threshold | Yes | Yes |
| Maximum area above/under threshold | Yes | No |
| Mean area above/under threshold | Yes | No |
