# Supplementary material for: Association of Continuously Measured Vital Signs With Respiratory Insufficiency in Hospitalized COVID-19 Patients: Retrospective Cohort Study
Source: Interact J Med Res. 2022 Nov 23;11(2):e40289. doi: 10.2196/40289 (PMC9688258; doi:10.2196/40289)
Supplement: Multimedia Appendix 2 [file ijmr_v11i2e40289_app2.docx]

| Multimedia Appendix 2. Baseline characteristics of patients stratified by availability of vital sign data. | | | | |
| --- | --- | --- | --- | --- |
|  | | All / heart rate (n=334) | Respiratory rate (n=288) | Oxygen saturation (n=238) |
| Age (median, IQR) | | 65 (55.3-73.8) | 65 (56-73.3) | 65 (56-74) |
| Male sex (n, %) | | 207 (62.0%) | 186 (64.5%) | 144 (60.5%) |
| Charlson Comorbidity Index (median, IQR) | | 0 (0-1) | 0 (0-1) | 0 (0-1) |
| Dexamethasone during admission (n, %) | | 262 (78.4%) | 224 (77.8%) | 198 (83.2%) |
| Diagnosed with pulmonary embolism (n, %) | | 23 (6.9%) | 18 (6.3%) | 20 (8.4%) |
| Treatment restrictions^a^ (n, %) | | 91 (27.2%) | 74 (25.7%) | 65 (27.3%) |
| Length of hospital stay (median, IQR) | | 7 (5-12) | 7 (5-12) | 8 (5-13) |
| ICU or MCU admission (n, %) | | 57 (17.1%) | 52 (18.0%) | 38 (16.0%) |
|  | Included in ‘resp insuf’ group^b^ | 27 (8.1%) | 25 (8.7%) | 22 (9.2%) |
| Mortality (n, %) | | 23 (6.9%) | 19 (6.6%) | 17 (7.1%) |
| Endpoint: respiratory insufficiency (n, %) | | 66 (19.7%) | 57 (19.8%) | 54 (22.7%) |
| IQR: inter quartile range, ICU: intensive care unit, MCU: medium care unit, resp insuf: combined endpoint of respiratory insufficiency. | | | | |
| ^a^Treatment restrictions: no resuscitation, no ventilation, and/or no ICU admission. | | | | |
| ^b^If a patient was monitored after ICU admission and did not reach the endpoint while being monitored, he/she was not included in the ‘respiratory insufficiency’ group. | | | | |
