# Supplementary material for: Association of Continuously Measured Vital Signs With Respiratory Insufficiency in Hospitalized COVID-19 Patients: Retrospective Cohort Study
Source: Interact J Med Res. 2022 Nov 23;11(2):e40289. doi: 10.2196/40289 (PMC9688258; doi:10.2196/40289)
Supplement: Multimedia Appendix 3 [file ijmr_v11i2e40289_app3.docx]

| **Multimedia Appendix 3. Mean of summary measures 4-hour timeframes.** | | | | | | | |
| --- | --- | --- | --- | --- | --- | --- | --- |
| Parameter | **Threshold** | **Summary measure** | **Total** | | **No resp insuf.** | | **Resp. insuf.** |
| Heartrate | **None** | Mean (/min) | 74.4 | | 71.7 | | 86.1 |
|  |  | Slope (/min/hour) | 0.04 | | -0.17 | | 0.99 |
|  |  | Variance (/min^2) | 53.6 | | 52.8 | | 56.7 |
|  | **>90/min** | Occurrence | 0.54 | | 0.48 | | 0.75 |
|  |  | Number of episodes | 2.47 | | 2.19 | | 3.66 |
|  |  | Total duration (min) | 37.5 | | 27.2 | | 81.6 |
|  |  | Total area above threshold | 907.2 | | 547.9 | | 2450.9 |
|  | **>110/min** | Occurrence | 0.19 | | 0.16 | | 0.35 |
|  |  | Number of episodes | 0.67 | | 0.49 | | 1.45 |
|  |  | Total duration (min) | 7.46 | | 4.24 | | 21.3 |
|  |  | Total area above threshold | 187.3 | | 89.9 | | 605.7 |
|  | **>130/min** | Occurrence | 0.037 | | 0.02 | | 0.12 |
|  |  | Number of episodes | 0.096 | | 0.035 | | 0.36 |
|  |  | Total duration (min) | 2.00 | | 0.98 | | 6.40 |
|  |  | Total area above threshold | 36.2 | | 22.5 | | 94.9 |
| Respiratory rate | **None** | Mean (/min) | 21.5 | | 20.5 | | 25.6 |
|  |  | Slope (/min/hour) | -0.04 | | -0.12 | | 0.30 |
|  |  | Variance (/min^2) | 5.46 | | 5.10 | | 6.94 |
|  | **>20/min** | Occurrence | 0.89 | | 0.88 | | 0.97 |
|  |  | Number of episodes | 4.15 | | 4.37 | | 3.23 |
|  |  | Total duration (min) | 124.9 | | 107.5 | | 196.5 |
|  |  | Total area above threshold | 622.0 | | 440.9 | | 1368.0 |
|  | **>24/min** | Occurrence | 0.65 | | 0.58 | | 0.93 |
|  |  | Number of episodes | 3.27 | | 2.75 | | 5.42 |
|  |  | Total duration (min) | 52.4 | | 33.4 | | 130.9 |
|  |  | Total area above threshold | 229.2 | | 125.5 | | 656.2 |
|  | **>29/min** | Occurrence | 0.25 | | 0.18 | | 0.56 |
|  |  | Number of episodes | 0.98 | | 0.55 | | 2.77 |
|  |  | Total duration (min) | 13.2 | | 5.88 | | 43.3 |
|  |  | Total area above threshold | 56.1 | | 25.14 | | 183.5 |
| Oxygen saturation | **None** | Mean (%) | 94.8 | | 95.2 | | 93.3 |
|  |  | Slope (%/hour) | -0.079 | | -0.030 | | -0.26 |
|  |  | Variance (%^2) | 5.28 | | 4.47 | | 8.19 |
|  | **<94%** | Occurrence | 0.81 | | 0.77 | | 0.98 |
|  |  | Number of episodes | 5.89 | | 5.24 | | 8.23 |
|  |  | Total duration (min) | 60.9 | | 47.7 | | 108.8 |
|  |  | Total area under threshold | 215.7 | | 159.0 | | 421.6 |
|  | **<92%** | Occurrence | 0.60 | | 0.51 | | 0.93 |
|  |  | Number of episodes | 3.12 | | 2.37 | | 5.84 |
|  |  | Total duration (min) | 24.5 | | 16.1 | | 55.0 |
|  |  | Total area under threshold | 95.4 | | 63.9 | | 210.0 |
|  | **<90%** | Occurrence | 0.40 | | 0.29 | | 0.79 |
|  |  | Number of episodes | 1.59 | | 1.16 | | 3.12 |
|  |  | Total duration (min) | 10.2 | | 6.16 | | 24.9 |
|  |  | Total area under threshold | 41.1 | | 25.7 | | 97.2 |
| Resp. insuf.: reached the combined endpoint of respiratory insufficiency | | | | | | | |
|  |  |  |  |  | |  | |

| Mean of summary measures 8-hour timeframes | | | | | | | |
| --- | --- | --- | --- | --- | --- | --- | --- |
| Parameter | **Threshold** | **Summary measure** | **Total** | | **No resp insuf.** | | **Resp. insuf.** |
| Heartrate | **None** | Mean (/min) | 75.0 | | 72.9 | | 84.5 |
|  |  | Slope (/min/hour) | -0.35 | | -0.59 | | 0.74 |
|  |  | Variance (/min^2) | 71.6 | | 70.8 | | 75.6 |
|  | **>90/min** | Occurrence | 0.67 | | 0.64 | | 0.80 |
|  |  | Number of episodes | 5.48 | | 5.18 | | 6.84 |
|  |  | Total duration (min) | 73.8 | | 58.1 | | 145.8 |
|  |  | Total area above threshold | 1574 | | 1058 | | 3946 |
|  | **>110/min** | Occurrence | 0.27 | | 0.24 | | 0.41 |
|  |  | Number of episodes | 1.20 | | 0.96 | | 2.33 |
|  |  | Total duration (min) | 13.22 | | 8.00 | | 37.2 |
|  |  | Total area above threshold | 271.2 | | 138.1 | | 882.1 |
|  | **>130/min** | Occurrence | 0.050 | | 0.028 | | 0.15 |
|  |  | Number of episodes | 0.14 | | 0.062 | | 0.48 |
|  |  | Total duration (min) | 2.92 | | 1.34 | | 10.2 |
|  |  | Total area above threshold | 41.5 | | 25.5 | | 115.0 |
| Respiratory rate | **None** | Mean (/min) | 21.6 | | 20.9 | | 25.1 |
|  |  | Slope (/min/hour) | -0.095 | | -0.17 | | 0.24 |
|  |  | Variance (/min^2) | 7.11 | | 6.65 | | 9.20 |
|  | **>20/min** | Occurrence | 0.96 | | 0.96 | | 0.99 |
|  |  | Number of episodes | 8.47 | | 8.91 | | 6.46 |
|  |  | Total duration (min) | 258.3 | | 231.3 | | 380.7 |
|  |  | Total area above threshold | 1254.4 | | 986.4 | | 2470 |
|  | **>24/min** | Occurrence | 0.78 | | 0.74 | | 0.95 |
|  |  | Number of episodes | 7.00 | | 6.27 | | 10.3 |
|  |  | Total duration (min) | 107.4 | | 79.1 | | 235.6 |
|  |  | Total area above threshold | 444.7 | | 291.2 | | 1141 |
|  | **>29/min** | Occurrence | 0.35 | | 0.29 | | 0.63 |
|  |  | Number of episodes | 2.06 | | 1.37 | | 5.17 |
|  |  | Total duration (min) | 24.7 | | 14.0 | | 73.2 |
|  |  | Total area above threshold | 96.6 | | 54.1 | | 289.3 |
| Oxygen saturation | **None** | Mean (%) | 95.0 | | 95.3 | | 93.6 |
|  |  | Slope (%/hour) | -0.046 | | -0.013 | | -0.17 |
|  |  | Variance (%^2) | 5.78 | | 5.14 | | 8.30 |
|  | **<94%** | Occurrence | 0.89 | | 0.86 | | 0.99 |
|  |  | Number of episodes | 11.2 | | 10.0 | | 15.6 |
|  |  | Total duration (min) | 124.2 | | 99.4 | | 220.3 |
|  |  | Total area under threshold | 378.4 | | 288.9 | | 726.2 |
|  | **<92%** | Occurrence | 0.71 | | 0.65 | | 0.98 |
|  |  | Number of episodes | 5.72 | | 4.45 | | 10.7 |
|  |  | Total duration (min) | 49.4 | | 33.1 | | 112.5 |
|  |  | Total area under threshold | 162.7 | | 115.9 | | 344.2 |
|  | **<90%** | Occurrence | 0.42 | | 0.52 | | 0.93 |
|  |  | Number of episodes | 2.08 | | 2.79 | | 5.54 |
|  |  | Total duration (min) | 13.43 | | 20.1 | | 46.1 |
|  |  | Total area under threshold | 47.3 | | 67.9 | | 148.0 |
| Resp. insuf.: reached the combined endpoint of respiratory insufficiency | | | | | | | |
|  |  |  |  |  | |  | |
